# A Rare Case of Small Bowel Obstruction Due to Sunflower Seeds Consumption in an Adult

**DOI:** 10.7759/cureus.14330

**Published:** 2021-04-06

**Authors:** Shirly Samuel, Artem Sharko, Jishna Shrestha, Robin Sherchan, Shaji Baig

**Affiliations:** 1 Internal Medicine, Northwestern Medicine McHenry Hospital, Rosalind Franklin University of Medicine and Science, McHenry, USA

**Keywords:** small bowel obstruction, bezoar, sunflower seeds bezoar, sunflower seeds obstruction, seed bezoar

## Abstract

Small bowel obstruction can occur due to multiple etiologies, including intra-abdominal adhesions, hernias, strictures, Crohn's disease, and malignancies. An infrequent cause of small bowel obstruction can be seed bezoars. We present a case of a 72-year-old male who presented to the emergency department with a clinical picture of small bowel obstruction without any apparent risk factors for this condition. After thorough questioning, it was revealed that he had consumed a large number of sunflower seeds prior to the onset of symptoms. This eventually was proved to be the cause of his symptoms. The case is presented with the intent to highlight the necessity of acquiring dietary history in patients with high suspicion for bowel obstruction without any predisposing risk factors.

## Introduction

Phytobezoars are formed due to the consumption of unshelled sunflower or pumpkin seeds, fruits with seeds, or kernels. When they occur, they can lead to constipation, obstruction, and ulcers as a result of intestinal mucosal damage [1]. Most reported cases involve pediatric patients with rectal bezoars causing fecal impaction [2]. It is rare for seed bezoars to cause obstruction of the small intestine, especially in adults [3]. We present a case of small bowel obstruction in an adult secondary to consumption of a large number of sunflower seeds.

## Case presentation

**Figure 1:**
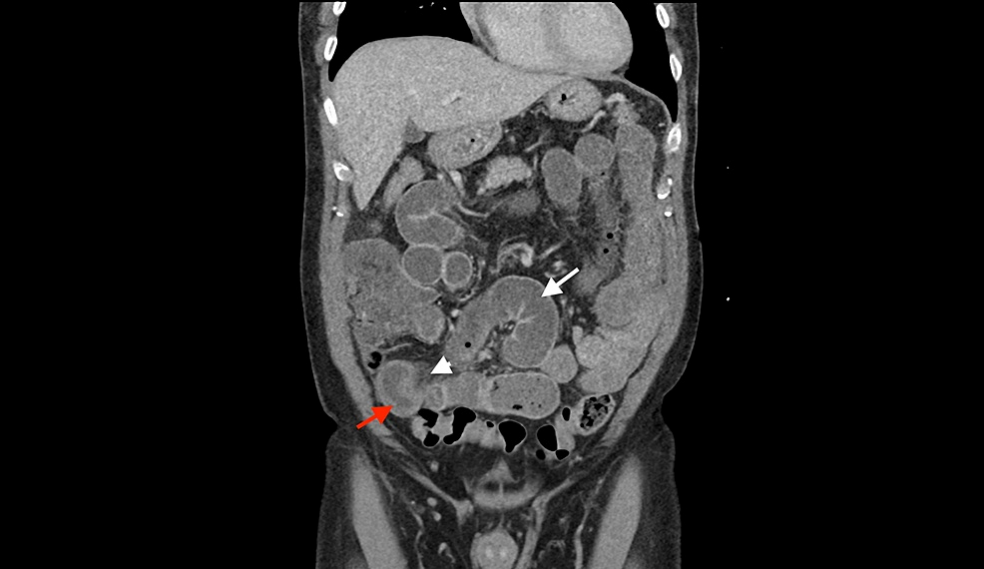
Computed tomography of the abdomen and pelvis: small bowel dilation measuring up to 3.5 cm in diameter (white arrow) with a transition point in the right lower quadrant where a wall thickening of the distal ileum (red arrow) is seen along with mesenteric fat stranding (white arrowhead). Findings are compatible with ileitis.

A 72-year-old male with a history of essential hypertension and without any history of abdominal surgeries presented to the emergency department with several hours of acute onset severe abdominal pain. The pain was colicky, non-radiating, and localized to the periumbilical area. He tried bismuth subsalicylate at home with no relief of symptoms. He had not passed any flatus and had no bowel movements since the onset of symptoms. He denied any fever, chills, diarrhea, nausea, or vomiting. He denied any past occurrences of similar episodes. On further questioning, he admitted to eating a large amount of fried, unshelled sunflower seeds the day before the symptoms started. He had a colonoscopy ten years ago which revealed a tortuous colon but no polyps or diverticulosis. There was no history of colon cancer or inflammatory bowel disease in the family. On presentation, vital signs were within normal limits. The abdominal exam revealed decreased bowel sounds, tenderness in the periumbilical area and right lower quadrant, no rebound tenderness was noted, and McBurney's sign was negative. All laboratory investigations, including a complete blood count, comprehensive metabolic panel, and lipase, were within normal limits. A CT of the abdomen was pertinent for a transition zone in the terminal ileum with wall thickening, fat stranding, and dilation of the bowel proximal to the transition zone (Figure 1). These findings were indicative of small bowel obstruction secondary to possible distal ileitis. The patient was managed conservatively with intravenous fluids and was kept nil per os.

**Figure 2:**
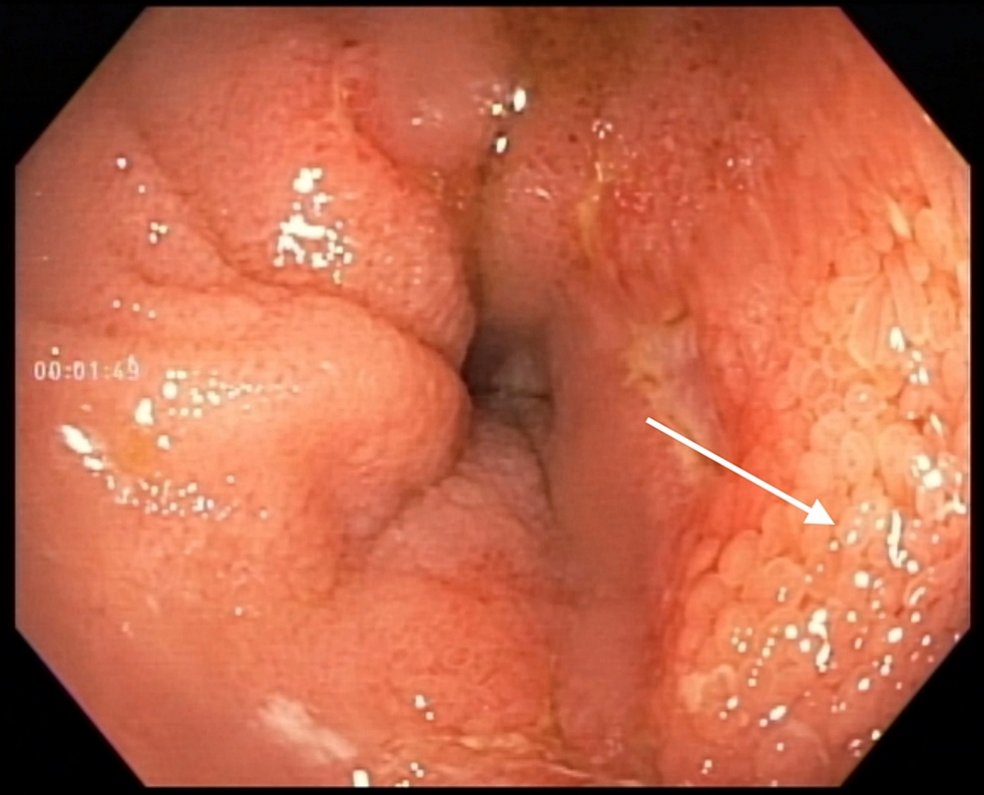
Colonoscopy image: nonspecific ulceration proximal to the ileocecal valve.

The next morning, the patient had two large volume bowel movements, after which his abdominal pain significantly improved. Colonoscopy was performed and showed nonspecific ulceration proximal to the ileocecal valve, suggestive of trauma, possibly from ingestion of a large number of sunflower seeds (Figure 2).

**Figure 3:**
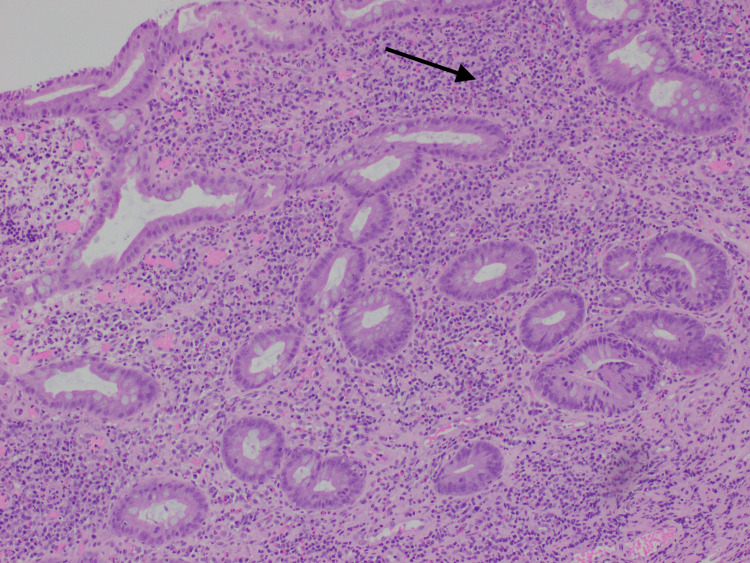
Hematoxylin and eosin stain showing mucosa of the terminal ileum with lymphoplasmacytic and neutrophilic infiltrates. No granulomas are present in the tissue.

At this point, early Crohn's disease was still in the differential. The pathology report showed focal surface epithelial erosion and florid lymphoplasmacytic and neutrophilic infiltrates. No granulomas were seen in the tissue (Figure 3). These findings favored traumatic ulceration, and Crohn's disease was ruled out. The patient continued to improve clinically with complete resolution of his abdominal pain.

## Discussion

Sunflower seeds are a popular dietary item for many people. There are several reported cases of children with small and large intestinal obstruction by phytobezoars containing rhubarb, raisins, or unshelled sunflower seeds. However, cases of complete or partial small bowel obstruction in adults are extremely rare [2]. According to a review of 153 cases of gastrointestinal seed bezoars, sunflower seeds were the second most common cause of obstruction, preceded only by watermelon seeds. Known risk factors for obstruction due to seed bezoars included consumption of high fiber products, prior gastric surgery, as well as poor chewing habits. The most common presenting symptom was constipation. Other symptoms included abdominal pain and rectal pain. The reviewers also noted that seed bezoars presenting as intestinal obstruction was seen in only 17% of cases, and these patients were found to have terminal ileum bezoars. The smallest diameter of the small bowel is located in the area proximal to the ileocecal valve. This area is also characterized by peristaltic waves that are weaker compared to the rest of the bowel [4]. This increases the risk of an obstruction in this location, as was seen in our case. It appears that in our case, the seeds were dislodged from the terminal ileum after temporarily causing obstruction, as evidenced by the large volume bowel movements that our patient had, as well as the fact that no bezoar was seen in the intestines during colonoscopy. In cases where the obstruction does not spontaneously resolve, small bowel enemas followed by evacuation via colonoscopy, fragmentation, as well as segmental enterotomy have been successfully utilized [4]. 

## Conclusions

Our patient had a partial small bowel obstruction caused by unshelled sunflower seeds, which resolved with conservative management. This case demonstrates that obtaining dietary history can aid in timely and accurate diagnosis in patients presenting with a clinical picture of bowel obstruction. Furthermore, educating patients about limiting the consumption of indigestible foods is essential as a prophylactic measure against such conditions.
